# Pervaporation Polyvinyl Alcohol Membranes Modified with Zr-Based Metal Organic Frameworks for Isopropanol Dehydration

**DOI:** 10.3390/membranes12100908

**Published:** 2022-09-20

**Authors:** Anna Kuzminova, Mariia Dmitrenko, Andrey Zolotarev, Danila Myznikov, Artem Selyutin, Rongxin Su, Anastasia Penkova

**Affiliations:** 1 St. Petersburg State University, 7/9 Universitetskaya nab., 199034 St. Petersburg, Russia; 2 State Key Laboratory of Chemical Engineering, School of Chemical Engineering and Technology, Tianjin University, Tianjin 300072, China

**Keywords:** mixed matrix membrane, polyvinyl alcohol, Zr-MOFs, pervaporation, isopropanol dehydration

## Abstract

Metal-organic frameworks (MOFs) are perceptive modifiers for the creation of mixed matrix membranes to improve the pervaporation performance of polymeric membranes. In this study, novel membranes based on polyvinyl alcohol (PVA) modified with Zr-MOFs (MIL-140A, MIL-140A-AcOH, and MIL-140A-AcOH-EDTA) particles were developed for enhanced pervaporation dehydration of isopropanol. Two membrane types (substrateless–freestanding; and formed on polyacrylonitrile support-composite) were prepared. The additional cross-linking of membranes with glutaraldehyde was carried out to circumvent membrane stability in pervaporation dehydration of diluted solutions. The synthesized Zr-MOFs were characterized by scanning electron microscopy, X-ray powder diffraction analysis, and specific surface area measurement. The structure and physicochemical properties of the developed membranes were investigated by Fourier-transform infrared spectroscopy, scanning electron and atomic force microscopies, thermogravimetric analysis, swelling experiments, and contact angle measurements. The PVA and PVA/Zr-MOFs membranes were evaluated in pervaporation dehydration of isopropanol in a wide concentration range. It was found that the composite cross-linked PVA membrane with 10 wt% MIL-140A had optimal pervaporation performance in the isopropanol dehydration (12–100 wt% water) at 22 °C: 0.15–1.33 kg/(m^2^h) permeation flux, 99.9 wt% water in the permeate, and is promising for the use in the industrial dehydration of alcohols.

## 1. Introduction

Membrane technologies for filtering [1,2,3,4] and separation [5,6,7,8,9] of various solvents, gas separation [10,11,12], sorption [13], etc. are becoming more and more widespread in research and technology. These sustainable processes have great advantages compared to conventional methods, such as equipment compactness, elimination of the use of additional toxic reagents, low energy consumption, etc. A membrane method pervaporation used for the separation of low molecular weight components is actively applied for the dehydration of alcohols, which is now a common task in many industries [14,15]. Water/isopropanol azeotropic mixture with 12 wt% water and a boiling point of 80.3 °C is the most common model water-alcohol mixture for membrane testing laboratories [16]. Recently, polymeric membranes have been widely used for this separation purpose, which have a large number of advantages over inorganic ones. A simple and effective way to improve the properties of polymeric membranes is their modification with various additives [17,18,19,20].

Metal-organic frameworks (MOFs) are porous structures that are composed of metal ions and polydentate organic molecules combined into a three-dimensional framework through strong metal-ligand interactions. MOFs have recently been actively used for various applications such as catalysis [21], chemical separation [22], adsorption [23], gas storage [24], drug delivery [25], and for the preparation of pervaporation membranes [26]. Mixed matrix membranes with MOFs as modifiers are promising for application in pervaporation due to the unique structural properties of MOFs, ease of modification, as well as good compatibility between MOFs and the polymer matrix. To develop pervaporation membranes, MOFs were used to modify polymers such as polyimide [27,28], cardo polyetherketone [29], polyether-block-amide [30], polyarylethersulfone [31], chitosan [32,33,34], a polymer of intrinsic microporosity PIM-1 [35], polydimethylsiloxane [36,37], polyamide [38], polyethyleneimine [39], sodium alginate [40,41], polyvinyl alcohol [42,43,44,45,46], etc.

The most widely used polymer for the dehydration of organic solvents is polyvinyl alcohol (PVA), due to its low cost, high hydrophilicity, chemical stability, excellent film-forming properties, and high tensile strength. A significant disadvantage of this polymeric material is its solubility in water, which does not allow the use of PVA membranes for separating the mixtures with high water content. For membrane stability in dilute solutions, cross-linking with various agents is used, such as citric or maleic acids [47], poly(sodium salt styrene sulfonic acid-co-maleic acid) [48], fumaric acid [49], urea-formaldehyde resin obtained by acid condensation [50], polyacrylic acid [51], glutaraldehyde [52,53,54], etc. For the development of PVA membranes with improved properties, such MOFs as UiO-66 [45,55], Cu_3_(BTC)_2_ [44], modified MIL-53(Al)-NH_2_ [46], SO_3_H-MIL-101-Cr [43], ZIF-8 [42,56,57], aluminum fumarate (AlFu) [58], etc. were tested. However, there is no information about membranes based on PVA modified with Zr-MOFs (MIL-140A, MIL-140A-AcOH, and MIL-140A-AcOH-EDTA) particles for pervaporation.

The metal ion has an important role in the stability of these synthesized MOFs. The rigid coordination nature of Lewis acids and bases of the Zr-carboxylate bonds makes MOFs more stable than, for instance, other high valence MOFs (for example, Fe-MOFs, etc.). In addition, Zr-MOFs are easily amenable to post-synthetic functionalization without loss of high stability, which makes them particularly attractive to researchers [59,60,61]. MIL-140A is Zr-MOF formed in the reactions of ZrCl_4_ with 1,4-benzenedicarboxylic acid (1,4-H_2_BDC) [62]. The functionalization of MIL-140A by acetic acid (AcOH) and ethylenediaminetetraacetic acid (EDTA) occurs by grafting AcOH and EDTA during synthesis. The introduction of functional groups into the MOF structure changes the shape and size of both particles and pores, causing the variation in porosity and specific surface area of Zr-MOFs. Unique properties of unmodified MIL-140A and modified MIL-140A-AcOH and MIL-140A-AcOH-EDTA particles, such as excellent thermal and chemical stability, high porosity, and tunable chemical properties may significantly and positively impact the characteristics of pervaporation PVA membranes.

The aim of the present work was to develop novel PVA membranes modified by Zr-MOFs (MIL-140A, MIL-140A-AcOH, and MIL-140A-AcOH-EDTA) with improved pervaporation performance (permeation flux and permeate composition) in isopropanol dehydration. The tailored membrane properties were achieved due to the unique physicochemical and structural properties of the Zr-MOFs modifier such as pore size and particle shape, thermal and chemical stability, large specific surface area and its ability to change the surface roughness, surface hydrophilic-hydrophobic balance, and swelling characteristics of PVA membranes. Two types of PVA and PVA/Zr-MOFs membranes (freestanding and composite, supported on polyacrylonitrile (PAN) substrate) were developed. The cross-linking of PVA-based membranes with glutaraldehyde (GA) was carried out to improve the stability in diluted aqueous solutions. The synthesized Zr-MOFs were studied by scanning electron microscopy (SEM), X-ray powder diffraction analysis (XRPD), and specific surface area measurement (BET). The structure of the developed membranes was investigated by Fourier-transform infrared spectroscopy (FTIR), scanning electron (SEM), and atomic force (AFM) microscopies. The changes in membrane physicochemical properties were studied by thermogravimetric analysis (TGA), swelling experiments, and contact angle measurements. The membrane performance of freestanding and composite membranes was evaluated in the pervaporation separation of water/isopropanol mixture in the wide concentration range.

## 2. Materials and Methods

### 2.1. Materials

Polyvinyl alcohol (PVA, molecular weight of 103 kDa, NevaReactiv, St. Petersburg, Russia) was used as a membrane material. Polyacrylonitrile (PAN, COA No.: A05P10833, the molecular weight of 150 kDa, Ming International Co., St. Petersburg, Russia) was used for the preparation of a porous substrate, because it showed good adhesion of the selective layer. MIL-140A (specific surface area of 493.4 ± 0.2 m^2^/g and pore diameter of 3.1 Å; Figure S3 in Supplementary Materials), MIL-140A-AcOH (specific surface area of 568.0 ± 0.1 m^2^/g and pore diameter of 4.4 Å; Figure S3 in Supplementary Materials) and MIL-140A-AcOH-EDTA (specific surface area of 529.3 ± 0.2 m^2^/g and pore diameter of 3.5 Å; Figure S3 in Supplementary Materials) were synthesized in the research group “Photoactive nanocomposite materials” at the Saint-Petersburg State University (St. Petersburg, Russia) and used for PVA modification (synthesis and characterization of Zr-MOFs particles are described in Supplementary Materials). The structure of the synthesized Zr-MOFs was confirmed by the X-ray powder diffraction method (XRPD) (Bruker “D8 DISCOVER”, Bruker, Billerica, Massachusetts, USA), shown in Figures S1 and S2 in Supplementary Materials [63]. Isopropanol (i-PrOH), dimethyl sulfoxide (DMSO), chloroform, and hydrochloric acid (36 wt%) (Vekton, St. Petersburg, Russia) were used without further purification. Glutaraldehyde (GA, 25 wt% aqueous solution, Sigma Aldrich, St. Petersburg, Russia) was used to cross-link the PVA-based membranes. Hydrochloric acid was used as a catalyst for cross-linking PVA chains with GA [64,65].

### 2.2. Freestanding Membrane Preparation

To develop unmodified membranes, a 2 wt% PVA solution was constantly stirred in water at 85 °C for 5 h. The PVA/Zr-MOFs composites were obtained by the solid-phase method with simultaneous mixing and grinding of PVA and Zr-MOFs powders in an agate mortar. Up to 15 wt% Zr-MOFs with respect to the PVA weight were added into the polymer matrix. The resulting PVA/Zr-MOFs composite was dissolved in water at 85 °C for 5 h with constant stirring. The obtained solutions of PVA and suspension of PVA/Zr-MOFs composites were sonicated at ambient temperature and cast into Petri dishes for the formation of membranes by solvent evaporation at 40 °C in an oven for 24 h. The thickness of the freestanding PVA and PVA/Zr-MOFs membranes measured with a micrometer was equal to 40 ± 5 μm.

To use the membranes in the separation of dilute solutions, the developed PVA and PVA/Zr-MOFs membranes were cross-linked with glutaraldehyde (GA). 25 wt% GA aqueous solution and 36 wt% hydrochloric acid were added into the PVA solution and PVA/Zr-MOFs suspension (0.033 mL on 1 g of PVA) [42,56] with subsequent stirring for 15 min. Then, the cross-linked freestanding membranes were prepared according to the procedure described above.

### 2.3. Composite Membrane Preparation

To prepare a porous substrate, 15 wt% PAN was dissolved in DMSO at 100–120 °C for 3 h with constant stirring using an overhead stirrer. Porous PAN substrate was prepared by phase inversion technique: the PAN solution was deposited with a casting blade (gap width of 200 µm) onto a glass support with the subsequent immersion in a coagulation bath with distilled water at ambient temperature (non-solvent induced phase separation-NIPS) [66].

The preparation of the composite cross-linked PVA and PVA/Zr-MOFs membranes was carried out as follows: to form a dense thin layer, PVA solution or PVA/Zr-MOFs suspension with GA and HCl were deposited onto a surface of porous PAN substrate stretched over a steel ring. Next, the ring was placed on the surface so that the composite membrane was perpendicular to the surface to runoff the excess polymer solution. The excess polymer was removed from the walls of the steel ring, and the membrane was dried on air for 24 h. The thickness of the selective layer measured by SEM was found to be 900 ± 50 nm.

### 2.4. Pervaporation Experiment

The membrane performance of the developed freestanding and composite PVA and PVA/Zr-MOFs membranes were studied in a laboratory pervaporation cell (the effective membrane area was 9.6 × 10^−4^ m^2^) with stirring in a stationary mode at 22, 50, and 70 °C [40]. The compositions of the feed and permeate were investigated using a gas chromatograph Chromatec Crystal 5000.2 (Chromatec, Nizhny Novgorod, Russia) with a column “Hayesep R” (2 m long and 3 mm in diameter) and a thermal conductivity detector.

The permeation flux *J* (kg/(m^2^h)) of the PVA and PVA/Zr-MOFs membranes was calculated by Equation (1) [67]:

(1) $$J=\frac{W}{A\cdot t},$$

where *W* (kg) is the weight of permeate (the mixture that permeated through the membrane), *A* (m^2^) is the effective membrane area (9.6 × 10^−4^ m^2^), and *t* (h) is the time of the measurement.

To ensure the accuracy of parameters, all the data were collected in triplicate, and the average value was used. The obtained average accuracies were as follows: ±0.5% for water content in the permeate, ±5% for permeation flux of the freestanding PVA and PVA/Zr-MOFs membranes, and ±3% for permeation flux of the composite PVA and PVA/Zr-MOFs membranes.

### 2.5. Fourier-Transform Infrared Spectroscopy

Structural changes of the freestanding PVA and PVA/Zr-MOFs membranes were studied by Fourier-transform infrared spectroscopy (FTIR) using IRAffinity-1S spectrometer (Shimadzu, St. Petersburg, Russia), to which an attenuated total reflectance (ATR) accessory was attached. The measurement was carried out in the range of 600–4000 cm^−1^ at 25 °C.

### 2.6. Atomic Force Microscopy

The surface topography of the PVA and PVA/Zr-MOFs membranes was studied by atomic force microscopy (AFM) using NT-MDT NTegra Maximus atomic force microscope (NT-MDT Spectrum Instruments, Moscow, Russia) with standard silicon cantilevers and rigidity of 15 N·m^−1^ in tapping mode.

### 2.7. Scanning Electron Microscopy

The cross-sectional and surface morphology of the PVA and PVA/Zr-MOFs membranes was studied by scanning electron microscopy (SEM) using Zeiss AURIGA Laser (Carl Zeiss SMT, Oberhochen, Germany) at 1 kV. A cross-section of the membranes was obtained by breaking the membrane in liquid nitrogen perpendicular to the surface.

### 2.8. Thermogravimetric Analysis

The thermochemical properties of the freestanding PVA and PVA/Zr-MOFs membranes were studied by thermogravimetric analysis (TGA) using Thermobalance TG 209 F1 Libra (Netzsch, Leuna, Germany) at a heating rate of 10 °C/min in argon atmosphere in the range of 30–586 °C.

### 2.9. Swelling Measurements

The swelling degree (sorption) was studied in water/isopropanol (12/88 and 30/70 wt%) mixtures for uncross-linked and cross-linked freestanding PVA and PVA/Zr-MOFs membranes, as well as in pure water for cross-linked freestanding PVA and PVA/Zr-MOFs membranes by the gravimetric method at 25 °C. Each membrane was lowered into a water/isopropanol mixture or water, and the weight of the membranes was checked regularly to the constant swelling weight.

To calculate swelling degree (*S*), Equation (2) was used:

(2) $$S=\frac{m_{s}-m_{o}}{m_{o}}\cdot 100\%,$$

where *m_s_* (g) is the weight of the swollen membrane, *m_o_* (g) is the initial weight of the dry membrane.

### 2.10. Contact Angle Measurements

To study the hydrophilic-hydrophobic surface balance of the cross-linked freestanding membranes, contact angles of water were measured using a Goniometer LK-1 (NPK Open Science Ltd., Krasnogorsk, Russia) by the sessile drop method. The contact angle data were calculated using the software “DropShape”.

### 2.11. Density Measurements

The density *ρ* (g/cm^3^) of the freestanding membranes was studied by flotation method at 22 °C. Chloroform (*ρ* = 1.49 g/cm^3^) and isopropanol (*ρ* = 0.78 g/cm^3^) were chosen as solvents to measure the density since they did not cause membrane swelling and did not react with them. The flotation method was carried out according to the methodology described earlier in ref. [68]. The density of each membrane was measured at least thrice, and the density of the solvent mixture was measured with a pycnometer.

## 3. Results and Discussion

Section 3 is divided into several subsections. Section 3.1 is devoted to the pervaporation performance, structural and physicochemical properties of the freestanding PVA and PVA/Zr-MOFs membranes: performance of uncross-linked membranes is presented in Section 3.1.1 (in this section, the influence of modifiers on pervaporation performance is studied), for cross-linked membranes-in Section 3.1.2 (in this section, the influence of cross-linking agent on pervaporation performance is studied), Section 3.1.3 contains physicochemical properties and structure investigation. Section 3.2 is devoted to the pervaporation performance, and structural properties of the composite PVA and PVA/Zr-MOFs membranes. Additionally, the synthesis and characterization of the developed Zr-MOFs particles are presented in Supplementary Materials: the investigation by X-ray powder diffraction (Figures S1 and S2), low-temperature nitrogen adsorption BET analysis (Figure S3), and scanning electron microscopy (Figure S4).

### 3.1. The Development and Investigation of the Freestanding PVA and PVA/Zr-MOFs Membranes

#### 3.1.1. Pervaporation Performance of the Uncross-Linked PVA and PVA/Zr-MOFs Membranes

**Figure 1:**
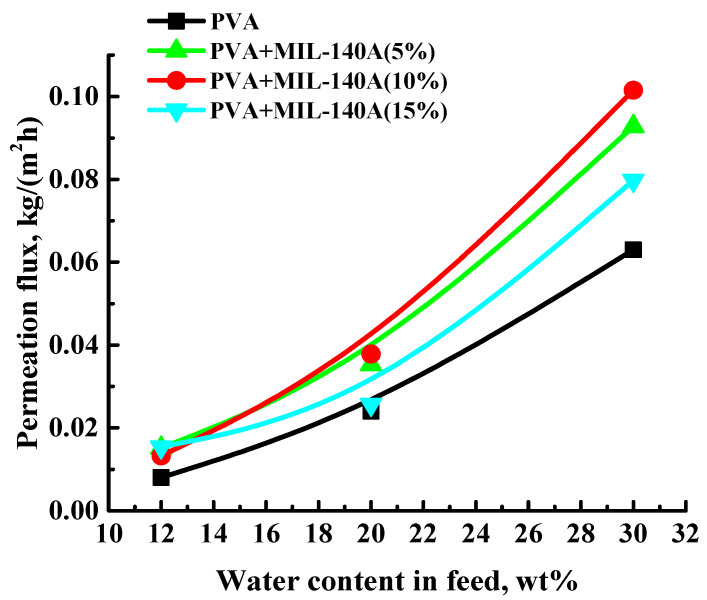
The dependence of the permeation flux on the water content in the feed for the uncross-linked PVA and PVA/MIL-140A membranes in pervaporation separation of water/isopropanol mixtures (12, 20, and 30 wt% water) at 22 °C. Water content in the permeate for all uncross-linked PVA and PVA/MIL-140A membranes was 99.9 wt%.

To optimize the concentration of Zr-MOFs in the polymer matrix, up to 15 wt% of unmodified Zr-MOF (MIL-140A) was introduced into the PVA matrix. The performance of the developed uncross-linked PVA and PVA/MIL-140A membranes were studied in pervaporation separation of water/isopropanol mixtures (12, 20, and 30 wt% water). The dependence of the permeation flux on the water content in the feed for the dehydration of isopropanol is shown in Figure 1.

The data presented in Figure 1 demonstrate that the permeation flux for the freestanding PVA and PVA/MIL-140A membranes increased with the rise of water content in the feed. This is related to the higher water concentration in the feed, resulting in increased swelling of the PVA-based membranes in the separated mixture (confirmed by the swelling data presented below). The introduction of MIL-140A (5–15 wt%) into the PVA matrix increased the permeation flux compared to the unmodified membrane that can be related to the formation of interfacial defects or gaps, the change of morphology and hydrophilic–hydrophobic surface balance which facilitated the penetrants diffusion [68]. The introduction of 5 wt% MIL-140A did not suffice to change significantly the performance of the PVA membrane, due to insignificant structural changes (SEM data presented below), surface roughness (AFM data presented below) and swelling degree (proven by swelling degree data presented below) during the modification. Further, 10 wt% of MIL-140A was shown to be the optimal concentration in the PVA matrix to get enhanced pervaporation performance of the PVA membrane, since PVA+MIL-140A(10%) membrane had the highest values of permeation flux due to morphology changes (SEM data presented below), increased surface roughness (AFM data presented below), and the highest swelling degree in water/isopropanol mixture among PVA and PVA+MIL-140A(5 and 15%) membranes (proven by swelling degree data presented below). The membrane modified with 10 wt% MIL-140A had ca. 1.6 times higher permeation flux than for the unmodified PVA membrane in pervaporation dehydration of isopropanol (30 wt% water). The increase in MIL-140A concentration to 15 wt% in the PVA matrix led to the formation of Zr-MOF agglomerates in the membrane (proven by SEM and AFM data presented below), hindering the penetration of components and decreasing the permeation flux. It should be noted that all membranes showed high selectivity with respect to water (99.9 wt% water in permeate).

**Figure 2:**
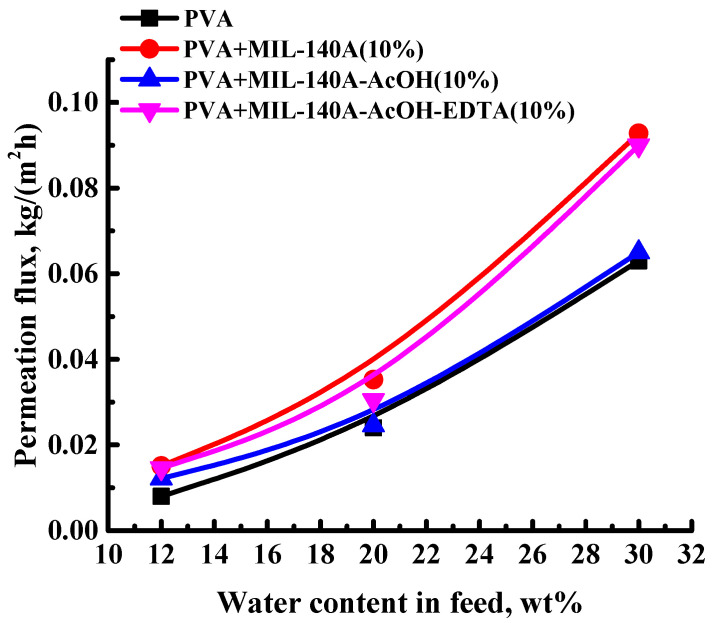
The dependence of the permeation flux on the water content in the feed for the uncross-linked PVA and PVA/Zr-MOFs(10%) membranes in pervaporation separation of water/isopropanol mixtures (12, 20, and 30 wt% water) at 22 °C. Water content in the permeate for all uncross-linked PVA and PVA/Zr-MOFs(10%) membranes was 99.9 wt%.

The addition of MOF without functional groups is known to possibly cause high permeability and low selectivity, due to the existence of interfacial defects. However, the functionalization of MOFs can decrease or exclude interface defects [69]. Thus, the effect of the introduction of functionalized MOFs (modified Zr-MOFs (MIL-140A-AcOH and MIL-140A-AcOH-EDTA)) was investigated. Based on the data presented in Figure 1, showing that 10 wt% MIL-140A is the optimal concentration, the same concentration of modified Zr-MOFs was added to compare MOFs’ properties to non-functionalized MIL-140A. The dependence of the permeation flux on the water content in the feed in pervaporation dehydration of isopropanol (12, 20, and 30 wt% water) is shown in Figure 2 for the developed uncross-linked PVA/MIL-140A-AcOH(10%) and PVA/MIL-140A-AcOH-EDTA(10%) membranes. Figure 2 also shows the permeation fluxes of the PVA and PVA/MIL-140A(10%) membranes for comparison.

The introduction of modified Zr-MOFs (10 wt%) into the PVA matrix increased the permeation flux, maintaining a high content of water in the permeate (99.9 wt%), compared to the pristine PVA membrane. The increased permeation flux of Zr-MOFs modified membranes was related to the change of membrane morphology, an increase in the swelling degree in water/isopropanol mixtures (12 and 30 wt% water), and surface roughness during the modification by the modified Zr-MOFs. The lowest values of permeation flux for all membranes were observed when separating an azeotropic water/isopropanol mixture, containing 12 wt% water, since the lowest values of the swelling degree were noted in the azeotropic mixture (proven by swelling degree data presented below). The membrane modified with MIL-140A (10 wt%) had the highest values of permeation flux compared to the pristine PVA and other modified PVA/Zr-MOFs(10%) membranes. The enhancement of the permeation flux for the PVA+MIL-140A(10%) membrane may be explained by the highest swelling in the separated mixture (confirmed by swelling data presented below) and surface roughness (confirmed by AFM data presented below) compared to the uncross-linked PVA and PVA/Zr-MOFs (PVA+MIL-140A-AcOH(10%) and PVA+MIL-140A-AcOH-EDTA(10%)) membranes. The introduction of AcOH and EDTA functional groups into the MOF structure increased pore size and specific surface area on the one hand (Figure S3 in Supplementary Materials) and altered particle shape and size on the other hand (Figure S4 in Supplementary Materials). Particle shape and size changes reduced membrane surface roughness (proven by AFM data presented below). Also, the modification with additional functional groups increased pore size in the particles, which could lead to the flow of the polymer into the pores of the MOF [70] and eliminate interfacial defects causing the decrease of the permeation flux. An increase in the specific surface area usually decreases the permeation flux [71,72,73,74]. Thus, the PVA+MIL-140A(10%) membrane was selected as optimal among the studied uncross-linked membranes.

#### 3.1.2. Pervaporation Performance of the Cross-Linked PVA and PVA/Zr-MOFs Membranes

**Figure 3:**
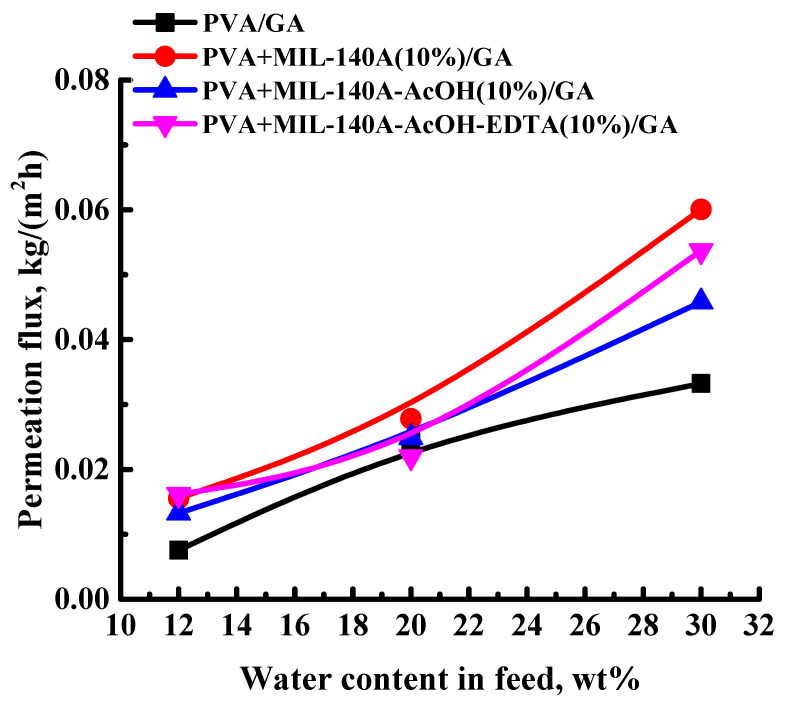
The dependence of the permeation flux on the water content in the feed for the cross-linked PVA and PVA/Zr-MOFs(10%) membranes in pervaporation separation of water/isopropanol mixtures (12, 20, and 30 wt% water) at 22 °C. Water content in the permeate for all cross-linked PVA and PVA/Zr-MOFs(10%) membranes was 99.9 wt%.

To implement freestanding membranes for the dehydration of mixtures with high water content, cross-linking of polymer chains with GA was applied. To study the effect of both modifiers (Zr-MOFs) and cross-linking agent (GA), the performance of the cross-linked freestanding PVA/GA and PVA/Zr-MOFs/GA membranes (PVA/GA, PVA+MIL-140A(10%)/GA, PVA+MIL-140A-AcOH(10%)/GA, and PVA+MIL-140A-AcOH-EDTA(10%)/GA) was tested in the pervaporation separation of water/isopropanol mixtures (12, 20, and 30 wt% water). The results are presented in Figure 3.

Cross-linking of membranes with GA results in a decrease in permeation flux compared to the corresponding uncross-linked membranes: ca. 1.9 times for the PVA/GA membrane, ca. 1.5 times for the PVA+MIL-140A(10%)/GA membrane, ca. 1.4 times for the PVA+MIL-140A-AcOH(10%)/GA membrane, ca. 1.7 times for the PVA+MIL-140A-AcOH-EDTA(10%)/GA membrane in pervaporation dehydration of isopropanol (30 wt% water). The decrease in permeation flux is related to the cross-linking of polymer chains, leading to a decrease in the free volume (confirmed by density data presented below) between them and resulting in the decreased swelling degree of the membranes in the separated mixture (confirmed by swelling data presented below). The optimum membrane performance, as in the case of the uncross-linked membranes, was exhibited by the membrane modified with MIL-140A (PVA+MIL-140A(10%)/GA membrane): permeation flux of 0.06 kg/(m^2^h), 99.9 wt% water in the permeate in pervaporation separation of the water/isopropanol mixture (30 wt% water). The enhancement of the permeation flux for this membrane may be related to the highest swelling in the separated mixtures (confirmed by swelling degree data presented below) and surface roughness (confirmed by AFM data presented below), compared to the cross-linked PVA/GA and PVA/Zr-MOFs/GA (PVA+MIL-140A-AcOH(10%)/GA and PVA+MIL-140A-AcOH-EDTA(10%)/GA) membranes. Thus, PVA+MIL-140A(10%)/GA membrane was found to be optimal among the cross-linked membranes for the dehydration of isopropanol.

#### 3.1.3. Structure and Physicochemical Properties of the Freestanding PVA and PVA/Zr-MOFs Membranes

***Fourier-transform infrared spectroscopy***

**Figure 4:**
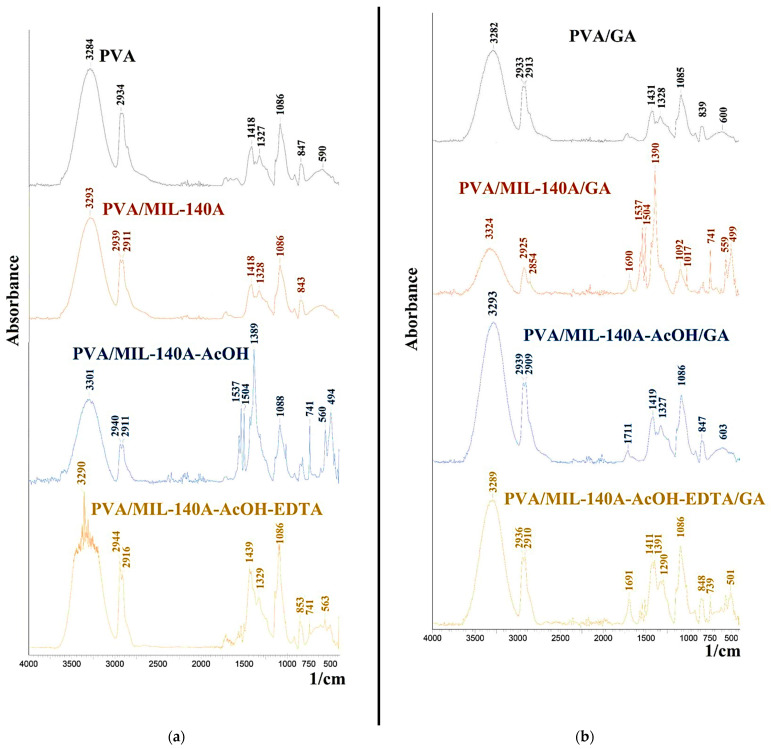
FTIR spectra of (**a**) the uncross-linked and (**b**) cross-linked PVA and PVA/Zr-MOFs(10%) membranes.

Fourier-transform infrared spectroscopy (FTIR) was used to study structural changes of the uncross-linked and cross-linked PVA and PVA/Zr-MOFs(10%) membranes (Figure 4).

The FTIR spectrum shown in Figure 4a for the PVA membrane shows a broad band at 3284 cm^−1^ and a peak with a maximum at 2934 cm^−1^, which correspond to vibrations of O-H and C-H bonds, respectively [75]. The absorption bands with maxima at 1327 and 1086 cm^−1^ correspond to vibrations associated with -C-O-H- group [75]. After the introduction of Zr-MOFs into the PVA matrix, FTIR spectra changed slightly. A shift of the peak at 3284 cm^−1^ for the PVA membrane to 3293 cm^−1^, 3301 cm^−1^, and 3290 cm^−1^ for the PVA+MIL-140A(10%), PVA+MIL-140A-AcOH(10%), and PVA+MIL-140A-AcOH-EDTA(10%) membranes, respectively, was noted. This shift may be attributed to the formation of hydrogen bonds between the -OH groups of PVA and the -OH and/or -COOH groups of Zr-MOFs [45].

The cross-linking of the PVA membrane with GA slightly changed the spectrum for the PVA/GA membrane (Figure 4b). There is a shift of the peak from 1418 cm^−1^ to 1431 cm^−1^, related to the deformation vibrations of the –OH groups; the decrease of band intensity in the region 3200–3350 cm^−1^ and the appearance of an intensive peak in the region 1650–1720 cm^−1^, referring to carbonyl groups [76]. The cross-linking of PVA chains with GA occurs with the formation of acetyl groups, according to the mechanism described in the study [76]. For the cross-linked PVA/Zr-MOFs/GA, the following changes in the FTIR spectra were noted: an increase in the intensity of the peak at 1690 cm^−1^ for the PVA/MIL-140A membrane, 1711 cm^−1^ for the PVA/MIL-140A-AcOH membrane and 1691 cm^−1^ for the PVA/MIL-140A-AcOH-EDTA membrane. These absorption bands may correspond to the stretching vibrations of C=O groups.

***Scanning electron microscopy***

**Figure 5:**
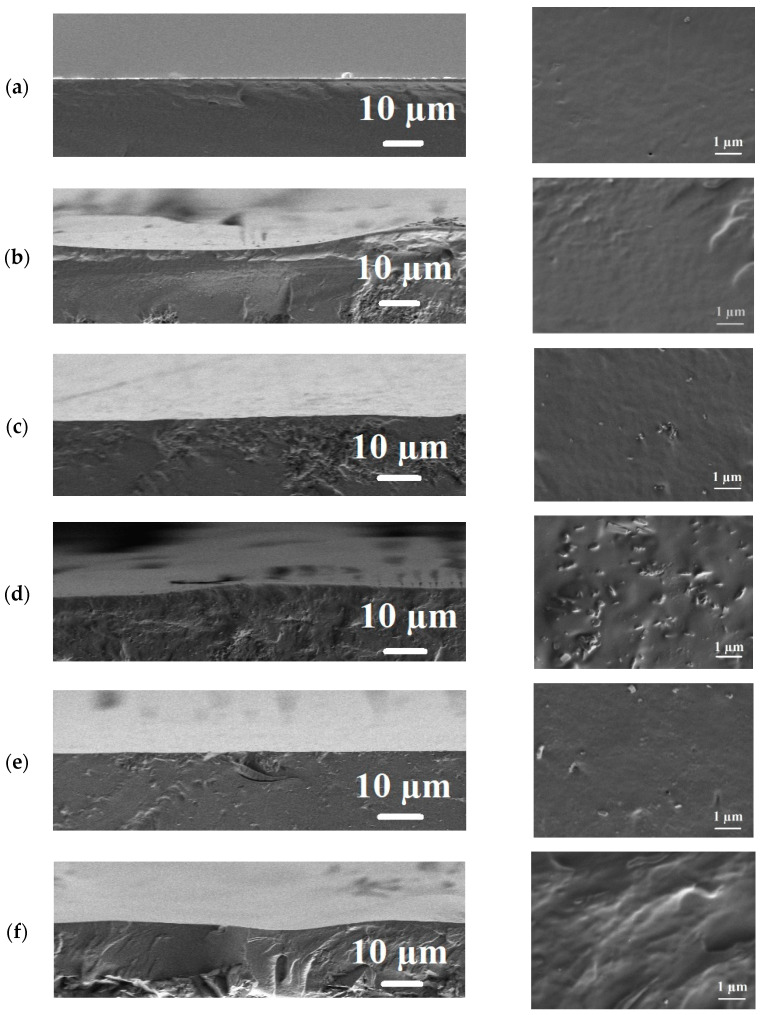
The cross-sectional SEM micrographs at different magnification for the uncross-linked PVA and PVA/Zr-MOFs membranes: (**a**) PVA; (**b**) PVA+MIL-140A(5%); (**c**) PVA+MIL-140A(10%); (**d**) PVA+MIL-140A(15%); (**e**) PVA+MIL-140A-AcOH(10%); and (**f**) PVA+MIL-140A-AcOH-EDTA(10%).

**Figure 6:**
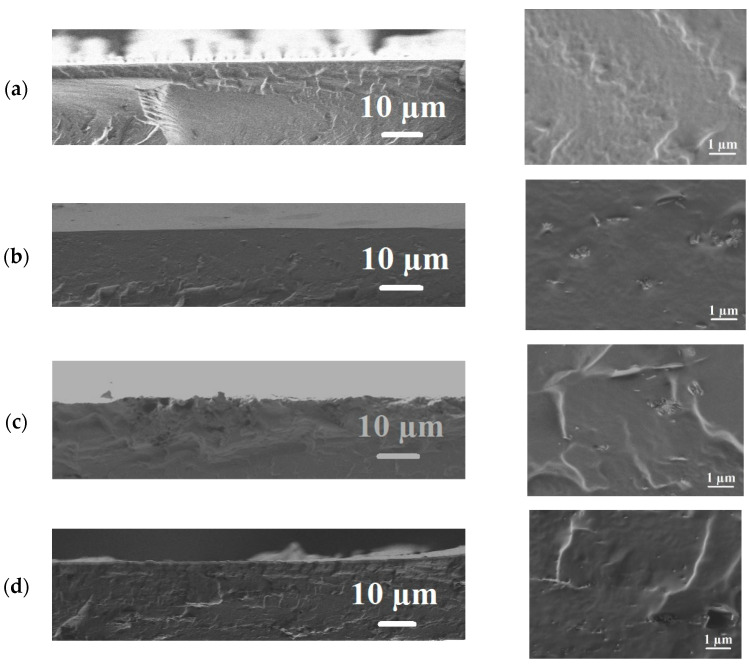
The cross-sectional SEM micrographs at different magnification for the cross-linked PVA and PVA/Zr-MOFs(10%) membranes: (**a**) PVA/GA; (**b**) PVA+MIL-140A(10%)/GA; (**c**) PVA+MIL-140A-AcOH(10%)/GA; and (**d**) PVA+MIL-140A-AcOH-EDTA(10%)/GA.

The inner structures of the uncross-linked and cross-linked PVA and PVA/Zr-MOFs membranes were studied by scanning electron microscopy (SEM). The cross-sectional SEM micrographs for the uncross-linked and cross-linked PVA and PVA/Zr-MOFs membranes are presented in Figure 5; Figure 6, respectively.

The presented SEM micrographs demonstrate that the uncross-linked PVA membrane had a rather smooth and uniform cross-sectional structure (Figure 5a). The introduction of 5 wt% MIL-140A (Figure 5b) slightly increased the roughness of the cross-section. The introduction of 10 and 15 wt% MIL-140A (Figure 5c,d) into the PVA matrix visualize MOF particles on the membrane cross-sections, the number of which increases with the rise of MIL-140A content. The introduction of 10 wt% MIL-140A-AcOH (Figure 5e) and MIL-140A-AcOH-EDTA (Figure 5f) into the PVA matrix also altered the cross-sectional structure of the membranes. These included: the appearance of plastic deformations, which are cross-sectional irregularities caused by immersion membrane in liquid nitrogen with subsequent cleavage and enhanced by embedded particles, and the visibility of particles Zr-MOFs, in particular MIL-140A-AcOH. The cross-sections of the modified membranes also differed depending on the introduced Zr-MOFs, due to the different shapes and structures (Figure S4 in Supplementary Materials) [69].

The cross-linking with GA created roughness on the cross-sectional structure. For cross-linked modified PVA/Zr-MOFs/GA membranes, Zr-MOFs particles are also visible on the membrane’s cross-sections. The changes in the inner morphology during the GA cross-linking and Zr-MOFs modification of the PVA were reflected significantly in the permeation fluxes of the developed membranes.

***Atomic force microscopy***

**Figure 7:**
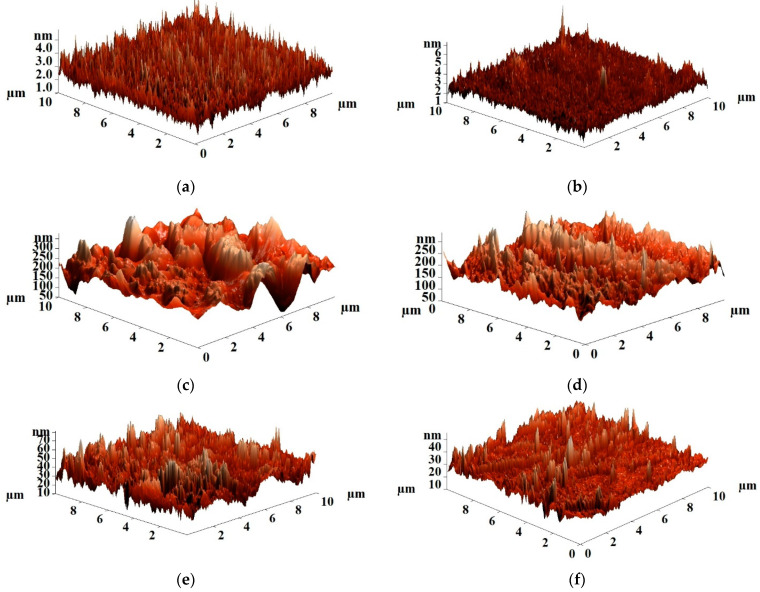
AFM images of the uncross-linked PVA and PVA/Zr-MOFs membranes: (**a**) PVA; (**b**) PVA+MIL-140A(5%); (**c**) PVA+MIL-140A(10%); (**d**) PVA+MIL-140A(15%); (**e**) PVA+MIL-140A-AcOH(10%); and (**f**) PVA+MIL-140A-AcOH-EDTA(10%).

**Figure 8:**
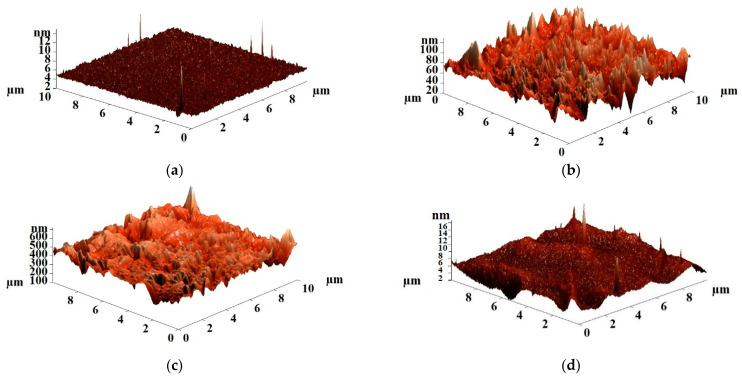
AFM images of the cross-linked PVA and PVA/Zr-MOFs(10%) membranes: (**a**) PVA/GA; (**b**) PVA+MIL-140A(10%)/GA; (**c**) PVA+MIL-140A-AcOH(10%)/GA; and (**d**) PVA+MIL-140A-AcOH-EDTA(10%)/GA.

The surface roughness of the uncross-linked and cross-linked PVA and PVA/Zr-MOFs membranes was studied by atomic force microscopy (AFM). AFM images with a scan size of 10 × 10 μm are presented in Figure 7; Figure 8 for the uncross-linked and cross-linked PVA and PVA/Zr-MOFs membranes, respectively.

The surface roughness characteristics (average, Ra, and root-mean-squared roughness, Rq) of the uncross-linked PVA and PVA/Zr-MOFs membranes were calculated based on the AFM images (Figure 7) and are presented in Table 1.

The data presented in Table 1 demonstrate that the introduction of Zr-MOFs into the PVA matrix increased surface average and root-mean-squared roughness. The increase of MIL-140A content in the PVA matrix from 5 to 15 wt% led to the rise of surface roughness characteristics. The PVA+MIL-140A(15%) membrane had the highest values of surface roughness due to the formation of particles agglomerates (confirmed by SEM data, Figure 5d). In comparison to the pristine PVA, PVA+MIL-140A-AcOH(10%) and PVA+MIL-140A-AcOH-EDTA(10%) membranes, the PVA+MIL-140A(10%) membrane had the highest surface roughness (Ra of 33.76 nm, Rq of 36.61 nm), which affects the facilitated sorption of feed components on the membrane surface (swelling degree data presented below), resulting to the highest permeation flux (Figure 2) among the uncross-linked PVA and PVA/Zr-MOFs(10%) membranes. The highest roughness values of this membrane among uncross-linked PVA, PVA+MIL-140A-AcOH(10%), and PVA+MIL-140A-AcOH-EDTA(10%) membranes can be associated with the MIL-140A shape, which is a narrow cylinder (Figure S4 in Supplementary Materials), and the smallest pore size [69].

Surface average (Ra) and root-mean-squared (Rq) roughness of the cross-linked PVA and PVA/Zr-MOFs(10%) membranes are presented in Table 2.

The cross-linking of PVA-based membranes with GA results in a reduction in values of surface average and root-mean-squared roughness compared to the uncross-linked membranes (Table 2). The dependence trend of the surface roughness of the cross-linked membranes is noted as in the case of the uncross-linked membranes (Table 1), which is in agreement with the obtained permeation fluxes (Figure 3). The cross-linked membrane modified with MIL-140A (PVA+MIL-140A(10%)/GA membrane) had the highest surface roughness values due to the particle pore size, as well as its shape, resulting in the maximum values of permeation flux among the cross-linked membranes.

***Thermogravimetric analysis***

**Figure 9:**
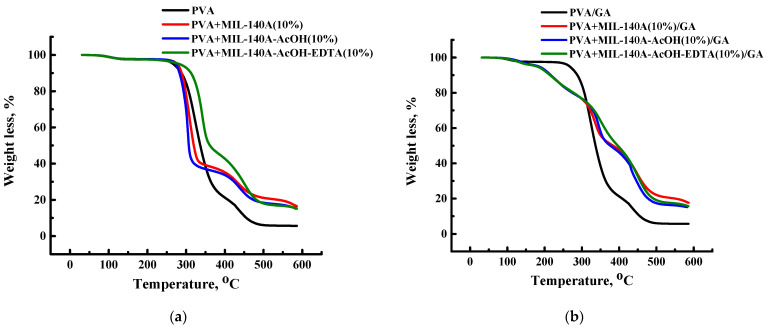
Thermogravimetric curves for (**a**) the uncross-linked and (**b**) cross-linked PVA and PVA/Zr-MOFs(10%) membranes.

The thermal stability of the uncross-linked and cross-linked PVA and PVA/Zr-MOFs(10%) membranes was investigated by thermogravimetric analysis (TGA). The obtained thermograms are presented in Figure 9.

Figure 9a shows three stages of weight loss for the uncross-linked PVA and PVA/Zr-MOFs(10%) membranes at the following temperature ranges: (1) 30–170 °C; (2) 170–398 °C; (3) >398 °C for the PVA membrane; (1) 30–178 °C; (2) 178–360 °C; (3) >360 °C for the PVA+MIL-140A(10%) membrane; (1) 30–159 °C; (2) 159–359 °C; (3) >359 °C for the PVA+MIL-140A-AcOH(10%) membrane; and (1) 30–168 °C; (2) 168–383 °C; (3) >383 °C for the PVA+MIL-140A-AcOH-EDTA(10%) membrane. The first stage is associated with the evaporation of water, which is present in the membranes, due to the absorption of atmospheric moisture. The weight loss for this step is approximately the same for all samples at 2.2–2.5%. The second step of weight loss is different for all samples: 78.5% at 398 °C for the PVA membrane, 61.5% at 360 °C for the PVA+MIL-140A(10%) membrane, 63.5% at 359 °C for the PVA+MIL-140A-AcOH(10%) membrane, 54.4% at 383 °C for the PVA+MIL-140A-AcOH-EDTA(10%) membrane. This step is attributed to the removal of hydroxyl groups attached to the polymer backbone. The last step of weight loss of samples refers to the decomposition of the membrane material. Wherein, the introduction of Zr-MOFs in the PVA matrix increased the thermal stability of membranes, the PVA+MIL-140A(10%) membrane has the highest thermal stability. The weight loss for the PVA membrane was 94.3%, while for the PVA+MIL140A(10%) membrane—80.5% at 550 °C.

There were four stages of weight loss for the cross-linked PVA/GA and PVA/Zr-MOFs(10%)/GA membranes at the following temperature ranges (Figure 9b): (1) 30–175°C; (2) 175–314 °C; (3) 314–513 °C; (4) >513 °C for the PVA/GA membrane; (1) 30–167 °C; (2) 167–279 °C; (3) 279–380°C; (4) >380 °C for the PVA+MIL-140A(10%)/GA membrane; (1) 30–170 °C; (2) 170–296 °C; (3) 296–379 °C; (4) >379 °C for the PVA+MIL-140A-AcOH(10%)/GA membrane; and (1) 30–164 °C; (2) 164–290 °C; (3) 290–398 °C; (4) >398 °C for the PVA+MIL-140A-AcOH-EDTA(10%)/GA membrane. The first area, as in the case of the uncross-linked membranes, was associated with the evaporation of water. The weight loss for this step was approximately the same for all samples being 2.5–4.4%. The following three steps differed significantly for the cross-linked PVA/GA membrane and cross-linked PVA/Zr-MOFs(10%)/GA membranes. The second step of weight loss was 26.6% at 314 °C for the unmodified PVA/GA membrane, 20.3–22.7% at 279–296 °C for PVA/Zr-MOFs(10%)/GA membranes and was attributed to the degradation of functional groups (for example, hydroxyl) attached to the polymer backbone. For the unmodified PVA/GA membrane, the weight loss was 94.1% at 513 °C, for PVA/Zr-MOFs(10%)/GA membranes the weight loss was 49.5–50.4% at the third step. The third step may correspond to the thermal decomposition of cross-linked PVA chains [77]. The final weight loss is related to the decomposition of the polymer’s main backbones and the decomposition of the PVA [78]. The PVA+MIL-140A(10%)/GA membrane had the highest thermal stability among the cross-linked membranes. The weight loss for the PVA/GA membrane was 94.3%, while for the PVA+MIL140A(10%)/GA membrane was 80.0% at 550 °C.

***Swelling degree***

The swelling of freestanding membranes was studied in water/isopropanol mixtures (12/88, 30/70 wt%) and water. For the uncross-linked PVA and PVA/Zr-MOFs membranes, the swelling degree is presented only for water/isopropanol (12/88, 30/70 wt%) mixtures, since these membranes instantly dissolve in pure water. The swelling data is shown in Table 3.

The data presented in Table 3 demonstrate that the addition of Zr-MOFs increased the swelling degree in the water/isopropanol (12/88 and 30/70 wt%) mixtures compared to the PVA and PVA/GA membranes. A slight increase in the swelling degree was caused by an increased number of sorption centers on the surface of the freestanding membranes. The introduction of MIL-140A (5 and 10 wt%) into the PVA matrix increased the swelling degree with the rise of its content in the membrane. However, the introduction of 15 wt% MIL-140A in the PVA membrane causes a slightly decreased swelling degree, compared to the PVA+MIL-140A(10%) membrane. It may be associated with the increased size of agglomerates and change of the membrane morphology, related to high modifier content (confirmed by SEM, Figure 5d). The introduction of 10 wt% modified Zr-MOFs (MIL-140A-AcOH and MIL-140A-AcOH-EDTA) increased the swelling compared to the PVA membrane but the swelling was slightly decreased compared to the MIL-140 modifier. The stability of PVA-based membranes in water was achieved by cross-linking with GA, as the membranes were found to be stable in water media for at least 14 days during the swelling study. Also, cross-linking of PVA chains resulted in a slight decrease in swelling degree in the water/isopropanol (12/88 and 30/70 wt%) mixtures compared to the uncross-linked membranes. The membranes modified with 10 wt% MIL-140A (PVA+MIL-140A(10%) and PVA+MIL-140A(10%)/GA) have the highest values of the swelling degree among the freestanding membranes based on PVA, PVA/MIL-140A-AcOH(10%), and PVA/MIL-140A-AcOH-EDTA(10%), which is in agreement with the highest surface roughness (Table 1 and Table 2) and permeation flux (Figure 2 and Figure 3) among them.

***Contact angle measurements***

To study changes in the surface properties of the PVA membrane during modification with Zr-MOFs, the contact angle of water for the cross-linked membranes was measured (Table 4). For the uncross-linked PVA and PVA/Zr-MOFs(10%) membranes, the contact angles were not possible to test since these membranes instantly dissolve in water.

The data presented in Table 4 demonstrate that modification of the membranes based on PVA with Zr-MOFs led to almost identical contact angle data. This may be explained by a number of factors that hampers a qualitative estimation of the changes in the hydrophilic-hydrophobic balance of the surface. These also include: changes in membrane roughness upon the introduction of a modifier with different functional groups which can also modulate membrane surface hydrophilicity.

***Density measurements***

To study the changes in the free volume of the PVA membrane during cross-linking and modification with 10 wt% Zr-MOFs, the density of the freestanding membranes were measured. The data are presented in Table 5.

The data presented in Table 5 demonstrate that the density of the PVA membrane was 1.26 g/cm^3^, which is comparable with the data presented in the literature [79]. The density of the PVA membranes modified with Zr-MOFs particles increased compared to the pristine PVA membranes, which was associated with the addition of MOF modifiers with a high density (density of Zr-MOFs ~0.5 g/cm^3^) [80]. Additionally, it can be associated with a denser polymer structure around the MOF particles [80] that caused the decreased free volume in the modified membranes.

### 3.2. The Development and Investigation of the Composite PVA and PVA/Zr-MOFs Membranes

**Figure 10:**
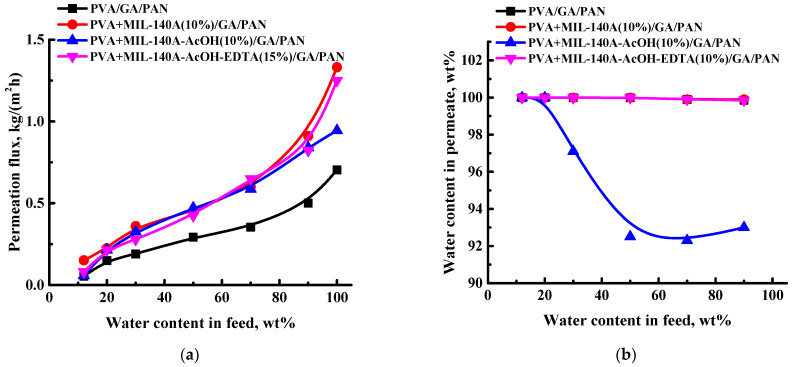
The dependence of the (**a**) permeation flux and (**b**) water content in permeate on the water content in the feed for the cross-linked composite PVA and PVA/Zr-MOFs(10%) membranes in pervaporation dehydration of isopropanol (12–100 wt% water) at 22 °C. The water content in the permeate for the PVA/GA/PAN, PVA+MIL-140A(10%)/GA/PAN, and PVA+MIL-140A-AcOH-EDTA(10%)/GA/PAN membranes was equal to ca. 99.9 wt%.

To increase the permeation flux of the cross-linked freestanding PVA/GA and PVA/Zr-MOFs(10%)/GA membranes for promising industrial application, composite membranes with thin selective layers were developed. Porous polyacrylonitrile membranes were used as a substrate, on which a thin selective cross-linked layer based on PVA/GA or PVA/Zr-MOFs(10%)/GA was deposited. The pervaporation performance of the cross-linked composite PVA and PVA/Zr-MOFs(10%) membranes (PVA/GA/PAN, PVA+MIL-140A(10%)/GA/PAN, PVA+MIL-140A-AcOH(10%)/GA/PAN, and PVA+MIL-140A-AcOH-EDTA(10%)/GA/PAN) in the pervaporation dehydration of isopropanol (12–100 wt% water) are presented in Figure 10.

Reducing the thickness of the selective layer by creating a composite membrane led to the rise of permeation flux and high content of water in the permeate. Thus, the permeation flux increased ca. three-fold in the permeation flux for the PVA/GA/PAN membrane compared to the uncross-linked PVA membrane, in the separation of a 30/70 wt% water/isopropanol mixture. For the modified PVA/Zr-MOFs(10%)/GA/PAN membranes, the increase in permeation flux compared to the uncross-linked membranes under the same conditions was as follows: ca. 3.9-fold for the PVA+MIL-140A(10%)/GA/PAN membrane, ca. 5-fold for the PVA+MIL-140A-AcOH(10%)/GA/PAN membrane, and ca. 3.1-fold for the PVA+MIL-140A-AcOH-EDTA(10%)/GA/PAN membrane. The selectivity of the membranes changed differently that depended on added Zr-MOF. Thus, for PVA/GA/PAN, PVA+MIL-140A(10%)/GA/PAN, and PVA+MIL-140A-AcOH-EDTA(10%)/GA/PAN membranes, the water content in the permeate was over 99.9 wt% in the pervaporation dehydration of isopropanol in the whole concentration range (12–90 wt% water). This is the reason for the coincidence of the dependence lines in Figure 10b. The decreased water content in the permeate (99.9–93 wt%) for the PVA+MIL-140A-AcOH(10%)/GA/PAN membrane may be associated with the specific MIL-140A-AcOH, that is, nature, pore size, shape, specific surface area, and the decreased thickness of a thin selective layer of the composite membrane compared to a freestanding membrane. Thus, the composite cross-linked PVA+MIL-140A(10%)/GA/PAN membrane had the optimal pervaporation performance, due to the highest permeation flux of 0.15–1.33 kg/(m^2^h) maintaining high water content in the permeate (99.9 wt% water) in pervaporation separation over the entire concentration range of the water/isopropanol mixture. This makes the developed pervaporation membrane promising for use in the industrial dehydration processes.

**Figure 11:**
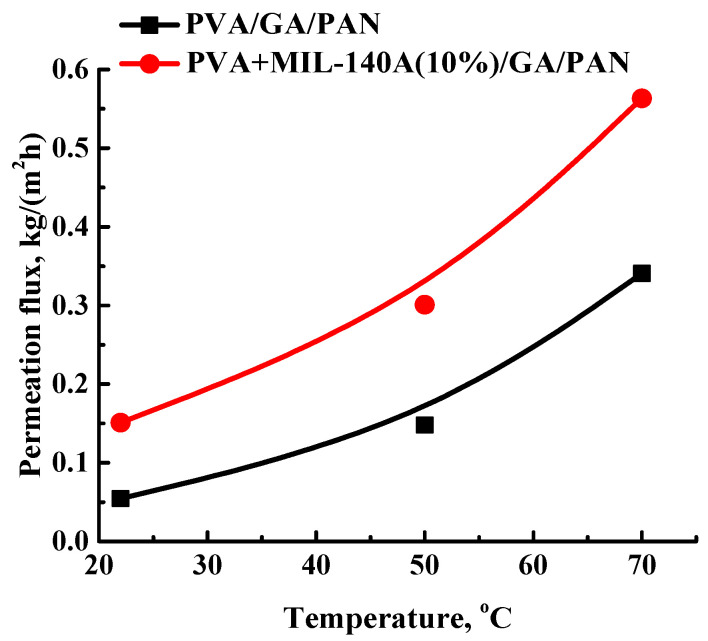
The dependence of the permeation flux on the temperature for cross-linked composite PVA/GA/PAN and PVA+MIL-140A(10%)/GA/PAN membranes in pervaporation separation of water/isopropanol mixture (12 wt% water). Water content in the permeate for PVA/GA/PAN and PVA+MIL-140A(10%)/GA/PAN membranes was 99.9 wt%.

To study the stability of the developed PVA+MIL-140A(10%)/GA/PAN membrane, the pervaporation separation of azeotropic water/isopropanol mixture was performed at different temperatures. The obtained results are presented in Figure 11. For comparison of the membrane performance, Figure 11 also shows the permeation flux of the PVA/GA/PAN membrane. 

It was found that with an increase in temperature, the permeation flux increased, which could be caused by the increase of the free volume in the matrix by increasing the movement of PVA chain segments [81]. The PVA+MIL-140A(10%)/GA/PAN membrane has a higher permeation flux at all temperatures tested, compared to the unmodified PVA/GA/PAN membrane because of the increased surface roughness, the changes of surface hydrophilic-hydrophobic membrane surface balance and interfacial defects or gaps [64]. The PVA+MIL-140A(10%)/GA/PAN membrane had a permeation flux of 0.563 kg/(m^2^h) and water content in permeate of 99.9 wt% in pervaporation separation of water/isopropanol mixtures (12 wt% water) at 70 °C.

**Figure 12:**
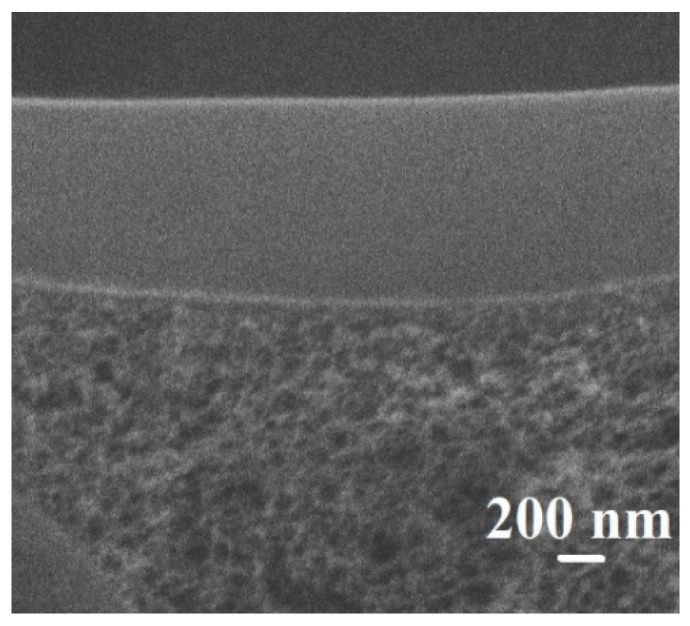
The cross-sectional SEM micrograph of the PVA/GA/PAN membrane.

The surface topography and morphology of the cross-linked composite PVA and PVA/Zr-MOFs(10%) membranes were studied by AFM and SEM. SEM cross-sectional micrograph of the PVA/GA/PAN membrane is presented in Figure 12, while the cross-sectional SEM micrographs of the cross-linked composite PVA/GA/PAN and PVA/Zr-MOFs(10%)/GA/PAN membranes were identical.

**Figure 13:**
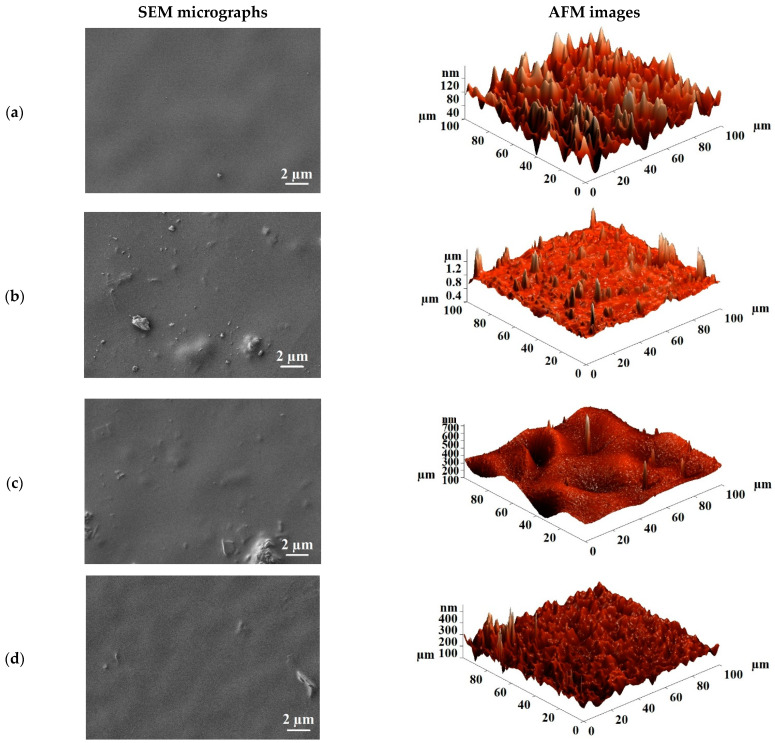
The AFM images and surface SEM micrographs for the cross-linked composite PVA and PVA/Zr-MOFs(10%) membranes: (**a**) PVA/GA/PAN; (**b**) PVA+MIL-140A(10%)/GA/PAN; (**c**) PVA+MIL-140A-AcOH(10%)/GA/PAN; and (**d**) PVA+MIL-140A-AcOH-EDTA(10%)/GA/PAN.

Good adhesion of the PVA/GA layer to the porous PAN substrate and uniform structure of the top thin selective layer is demonstrated. The thickness of the layer is ca. 900 nm. The AFM images with a scan size of 100 × 100 μm and surface SEM micrographs for the cross-linked composite PVA and PVA/Zr-MOFs(10%) membranes are presented in Figure 13.

On the surface SEM micrographs of the developed composite modified PVA/Zr-MOFs(10%)/GA/PAN membranes (Figure 13) Zr-MOFs particles can be observed. The presence of the particles causes the increased surface roughness and the changes in surface hydrophilic-hydrophobic balance, due to specific Zr-MOFs nature, pore size, shape, and specific surface area, as well as due to the formation of interfacial defects [69].

The surface roughness characteristics (average, Ra, and root-mean-squared, Rq, roughness) of the cross-linked composite PVA/GA/PAN and PVA/Zr-MOFs(10%)/GA/PAN membranes were calculated based on the AFM images (Figure 13) and presented in Table 6.

The data presented in Table 6 demonstrate that the values of surface roughness are higher for modified PVA/Zr-MOFs(10%)/GA/PAN membranes compared to the unmodified PVA/GA/PAN membrane, as in the case of the freestanding membranes, and in agreement with the obtained pervaporation performance for composite membranes (Figure 10). The PVA+MIL-140A(10%)/GA/PAN membrane had the maximum values of surface average (Ra) and root-mean-squared roughness (Rq), which was consistent with the highest permeation flux among the cross-linked composite PVA/GA/PAN and PVA/Zr-MOFs(10%)/GA/PAN membranes, due to facilitated sorption of the feed components.

### 3.3. Comparison of the Performance with PVA-Based Membranes

The comparison of the pervaporation performance of the cross-linked composite PVA+MIL-140A(10%)/GA/PAN membrane to the PVA-based membranes and the commercial analogue PERVAP^TM^ 1201 membrane (Sulzer Chemtech, Switzerland) described in the literature for the pervaporation dehydration of isopropanol under close experimental conditions are presented in Table 7.

The developed cross-linked composite PVA+MIL-140A(10%)/GA/PAN membrane was demonstrated to have improved membrane performance (increased permeation and/or water content in the permeate) in the pervaporation dehydration of isopropanol (20 and 30 wt% water) compared to the PVA-based membranes described in the literature. The membranes PVA+Pluronic F127 (3 wt%) cross-linked with maleic acid deposited on polyamide (17 wt%) support [86] and PVA+Graphene oxide quantum dots (GOQDs) (300 ppm) [87] had higher permeation flux and slightly lower water content in the permeate (97.7 and 99.5 wt%, respectively) compared to the developed PVA+MIL-140A(10%)/GA/PAN membrane in the pervaporation dehydration of isopropanol (30 wt% water). It should be also mentioned that this developed membrane has 6.6 and 12.8 times higher permeation flux than the commercial analogue PERVAP™ 1201 membrane in the separation of a water/isopropanol mixture with 20 and 30 wt% of water, respectively. This demonstrated the promising application of the developed cross-linked composite PVA+MIL-140A(10%)/GA/PAN membrane in the industrial dehydration processes.

## 4. Conclusions

In the present study, novel mixed matrix PVA membranes modified by Zr-MOFs (MIL-140A, MIL-140A-AcOH, and MIL-140A-AcOH-EDTA) with enhanced pervaporation performance were developed for dehydration of isopropanol. The improvement of PVA-based membrane characteristics was achieved due to the use of parent MIL-140A and functionalized MOF (MIL-140A-AcOH, and MIL-140A-AcOH-EDTA) that had different pore size, shape, and specific surface area. It allowed changing the membrane surface roughness, a hydrophilic-hydrophobic balance, swelling characteristics, thermal properties, and membrane performance.

Two kinds of freestanding PVA and PVA/Zr-MOFs membranes were prepared: uncross-linked and cross-linked. Cross-linking by GA was applied to increase the stability of these membranes in contact with dilute aqueous solutions. For both freestanding membranes kinds, the highest permeation flux was observed in the case of the introduction of 10 wt% MIL-140A into the PVA matrix, which could be explained by the highest swelling degree, surface roughness, and hydrophilicity compared to other membranes modified with 10 wt% functionalized Zr-MOFs (confirmed by swelling, and AFM data). Moreover, this membrane had the highest thermal stability (confirmed by TGA and pervaporation experiment at the elevated temperature of 50 and 70 °C).

To improve the performance of the freestanding cross-linked PVA/Zr-MOFs membranes in pervaporation separation of water/isopropanol mixture, the thickness of the selective layer was reduced by developing the cross-linked composite membranes on a PAN substrate. It was found that among composite cross-linked membranes, composite cross-linked PVA membrane with 10 wt% MIL-140A also had the highest membrane performance in the pervaporation dehydration of isopropanol (12–100 wt% water) at 22 °C: permeation flux of 0.15–1.33 kg/(m^2^h), 99.9 wt% water content in the permeate. This membrane is promising for use in the industrial dehydration of alcohols. 

## Figures and Tables

**Table 1:** The values of surface average (Ra) and root-mean-squared (Rq) roughness of the uncross-linked PVA and PVA/Zr-MOFs membranes.

Membranes	Ra, nm	Rq, nm
PVA	0.38	0.68
PVA+MIL-140A(5%)	1.79	2.52
PVA+MIL-140A(10%)	33.76	36.61
PVA+MIL-140A(15%)	41.89	52.89
PVA+MIL-140A-AcOH(10%)	13.54	17.71
PVA+MIL-140A-AcOH-EDTA(10%)	14.62	21.01

**Table 2:** The values of surface average (Ra) and root-mean-squared surface (Rq) roughness of cross-linked PVA and PVA/Zr-MOFs(10%) membranes.

Membranes	Ra, nm	Rq, nm
PVA/GA	0.35	1.31
PVA+MIL-140A(10%)/GA	19.24	24.72
PVA+MIL-140A-AcOH(10%)/GA	10.54	15.21
PVA+MIL-140A-AcOH-EDTA(10%)/GA	14.64	21.84

**Table 3:** Swelling degree of the uncross-linked and cross-linked PVA and PVA/Zr-MOFs membranes in water and water/isopropanol (12/88 and 30/70 wt%) mixtures.

Membrane	Swelling Degree, %		
	Water/Isopropanol Mixture		Water
	12/88 wt%	30/70 wt%	
PVA	15	53	-
PVA+MIL-140A(5%)	18	58	-
PVA+MIL-140A(10%)	20	61	-
PVA+MIL-140A(15%)	19	58	-
PVA+MIL-140A-AcOH(10%)	16	52	-
PVA+MIL-140A-AcOH-EDTA(10%)	17	53	-
PVA/GA	14	50	236
PVA+MIL-140A(10%)/GA	19	56	293
PVA+MIL-140A-AcOH(10%)/GA	15	51	248
PVA+MIL-140A-AcOH-EDTA(10%)/GA	16	52	250

**Table 4:** The contact angle of water for the cross-linked PVA and PVA/Zr-MOFs(10%) membranes.

Membranes	Contact Angle of Water, °
PVA/GA	67
PVA+MIL-140A(10%)/GA	64
PVA+MIL-140A-AcOH(10%)/GA	66
PVA+MIL-140A-AcOH-EDTA(10%)/GA	65

**Table 5:** Density of the freestanding uncross-linked and cross-linked PVA and PVA/Zr-MOFs(10%) membranes.

Membrane	Density, g/cm^3^
PVA	1.26
PVA+MIL-140A(10%)	1.31
PVA+MIL-140A-AcOH(10%)	1.29
PVA+MIL-140A-AcOH-EDTA(10%)	1.30
PVA/GA	1.27
PVA+MIL-140A(10%)/GA	1.32
PVA+MIL-140A-AcOH(10%)/GA	1.31
PVA+MIL-140A-AcOH-EDTA(10%)/GA	1.30

**Table 6:** The values of surface average (Ra) and root-mean-squared (Rq) roughness of the cross-linked composite PVA/GA/PAN and PVA/Zr-MOFs(10%)/GA/PAN membranes.

Membranes	Ra, nm	Rq, nm
PVA/GA/PAN	18.07	22.77
PVA+MIL-140A(10%)/GA/PAN	45.01	78.88
PVA+MIL-140A-AcOH(10%)/GA/PAN	37.51	51.35
PVA+MIL-140A-AcOH-EDTA(10%)/GA/PAN	26.04	34.55

**Table 7:** Pervaporation performance of the developed cross-linked composite PVA+MIL-140A(10%)/GA/PAN membrane and literature-described PVA-based membranes applied for dehydration of isopropanol.

Membranes	Thickness, μm	Water Content in the Feed, wt%	Temperature, °C	Permeation Flux, g/(m^2^h)	Water Content in Permeate, wt%	Reference
PVA+MIL-140A(10%)/GA/PAN	0.9	20	22	225	99.9	This study
PERVAP^TM^ 1201	-	20	22	34	99.9	[75]
PVA+cellulose nanofiber (6 wt%)	53-54	20	40	~65	~99.9	[82]
PVA+Polydopamine coated halloysite nanotube (5 wt%)	~70	20	40	190	~99.2	[83]
PVA+PVAm (polyvinyl amine)+Surface-modified halloysite nanotube (5 wt%)	75	20	40	130	~99.1	[84]
PVA+poly(acrylic acid)+Ag-modified zeolite incorporation (12.5 g)	50	20	40	84	~99.9	[85]
PVA+MIL-140A(10%)/GA/PAN	0.9	30	22	360	99.9	This study
PERVAP^TM^ 1201	-	30	22	28	~99.9	[40]
PVA+ Pluronic F127 (3 wt%) cross-linked with maleic acid deposited on polyamide (17 wt%) support	1.5	30	22	620	97.7	[86]
PVA/hydroxyethyl cellulose (70/30 wt%)+ carboxyfullerene (5 wt%)	30	30	22	193	~98.7	[68]
PVA+Graphene oxide quantum dots (GOQDs) (300 ppm)	3	30	25	463.5	~99.5	[87]

## Data Availability

Not applicable.

## Supplementary Materials

The following supporting information can be downloaded at: https://www.mdpi.com/article/10.3390/membranes12100908/s1, Figure S1: XRPD of the synthesized MIL-140A; Figure S2: Shifted XRPD of the synthesized modified Zr-MOFs: MIL-140A-AcOH (red line), MIL-140A-AcOH-EDTA (blue line), simulated from cif (black line).; Figure S3: Low temperature N_2_ adsorption isotherms: MIL-140A (green line), MIL-140A-AcOH (red line), MIL-140A-AcOH-EDTA (blue line).; Figure S4: SEM images of the (a) MIL-140A, (b) MIL-140A-AcOH, and (c) MIL-140A-AcOH-EDTA.
