# Supplementary material for: Pervaporation Polyvinyl Alcohol Membranes Modified with Zr-Based Metal Organic Frameworks for Isopropanol Dehydration
Source: Membranes (Basel). 2022 Sep 20;12(10):908. doi: 10.3390/membranes12100908 (PMC9611522; doi:10.3390/membranes12100908)
Supplement: Supplementary file 1 [file membranes-12-00908-s001.zip › membranes-1888277-supplementary.pdf]

membranes

Supplementary Information

Pervaporation Polyvinyl Alcohol Membranes Modified with Zr-Based Metal Organic Frameworks for Isopropanol Dehydration

Anna Kuzminova [1,*], Mariia Dmitrenko [1], Andrey Zolotarev [1], Danila Myznikov [1], Artem Selyutin [1], Rongxin Su [2] and Anastasia Penkova [1]

[1] St. Petersburg State University, 7/9 Universitetskaya nab., 199034 St. Petersburg, Russia
[2] State Key Laboratory of Chemical Engineering, School of Chemical Engineering and Technology, Tianjin University, Tianjin 300072, China
* Correspondence: a.kuzminova@spbu.ru; Tel.: +7(812)363-60-00 (ext. 3367)

Citation: Kuzminova, A.; Dmitrenko, M.; Zolotarev, A.; Myznikov, D.; Selyutin, A.; Su, R.; Penkova, A. Pervaporation Polyvinyl Alcohol Membranes Modified with Zr-Based Metal Organic Frameworks for Isopropanol Dehydration. *Membranes* **2022**, *12*, 908. https://doi.org/10.3390/membranes12100908

Academic Editors: Harsh Vardhan and Francis Verpoort

Received: 12 August 2022
Accepted: 14 September 2022
Published: 20 September 2022

Publisher's Note: MDPI stays neutral with regard to jurisdictional claims in published maps and institutional affiliations.

Copyright: © 2022 by the authors. Submitted for possible open access publication under the terms and conditions of the Creative Commons Attribution (CC BY) license (https://creativecommons.org/licenses/by/4.0/).

S1. Materials

$C_8H_6O_4$ (1,4-benzdicarboxylic acid, terephthalic acid, BDC, Sigma-Aldrich), $ZrCl_4$ (Zirconium(IV) chloride, Sigma-Aldrich), $C_2H_4O_2$ (Acetic acid anhydride, AcOH, 99.99%, Vecton), $C_{10}H_{14}N_2Na_2O_8{\cdot}2H_2O$ (Ethylenediaminetetraacetic acid disodium salt dihydrate, EDTA-Na_2, Panreac Qiumica S.A.U.), C_3H_7NO (N,N-Dimethylformamide, DMF, Vekton), CH_3OH (Methanol, MeOH, Vekton) were used without additional purification.

S2. Zr-MOFs preparation

MIL-140A

MIL-140A was synthesized by the following procedure: 6.4 mmol of 1,4-benzdicarboxylic acid and 6.4 mmol of zirconium chloride were dissolved in 100 mL of N,N-dimethylformamide (DMF). The obtained solution was placed in a Teflon autoclave and heated up to 150°C. The reaction mixture was withstood for 24 h under this temperature. Then, the mixture was cooled down to room temperature, and the obtained precipitate was filtered and washed with DMF three times. After this, the powder was dried at 100°C under vacuum and immersed in MeOH for 20 min with the following centrifugation. The last step was repeated two times more. The resulting product was dried at 80 °C under vacuum conditions.

MIL-140A-AcOH

The BDC (6.4 mmol), $ZrCl_4$ (6.4 mmol), and 1.5 mL of AcOH were dissolved consistently in 100 mL of DMF. The solution was placed in a Teflon autoclave and heated up in an oven at 150°C for 24 hours. The resulting precipitate was filtered and washed with DMF three times. After this, the powder was dried at 100°C under vacuum and immersed in MeOH for 20 min with the following centrifugation. The last step was repeated two times more. The resulting product was dried at 80°C under vacuum conditions.

MIL-140A-AcOH-EDTA

The obtained MIL-140A-AcOH (1.12 g) was dispersed in a solution of EDTANa_2 (27.91 mmol) in H2O (100 mL). The obtained mixture was sonicated for 20 min and placed in a Teflon autoclave, heating at 60°C for 12 hours. The resulting product was washed with water three times and dried at 70°C under vacuum.

S3. Zr-MOFs investigation

The X-ray powder diffraction

Crystal structure of the synthesized Zr-MOFs was studied by the X-ray powder diffraction method (XRPD) using a Bruker "D8 DISCOVER" high-resolution diffractometer with Cu Kα radiation over the 2Θ range of 5–60° for MIL-140A, 5–70° for MIL-140A-AcOH and MIL-140A-AcOH-EDTA with scan-steps of 0.03°. XRPD patterns of Zr-MOFs are presented in Figures S1 and S2.

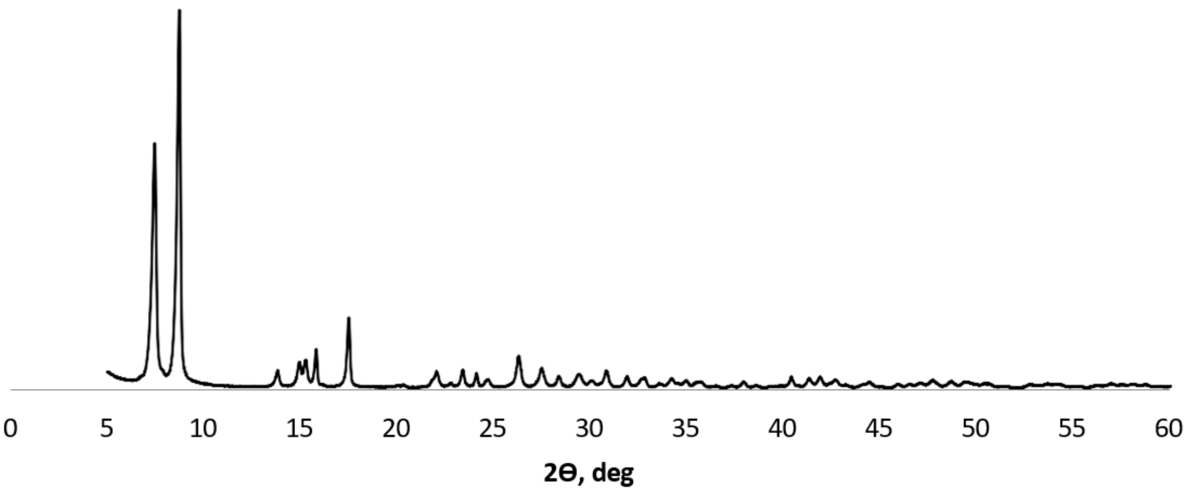

Figure S1. XRPD of the synthesized MIL-140A.

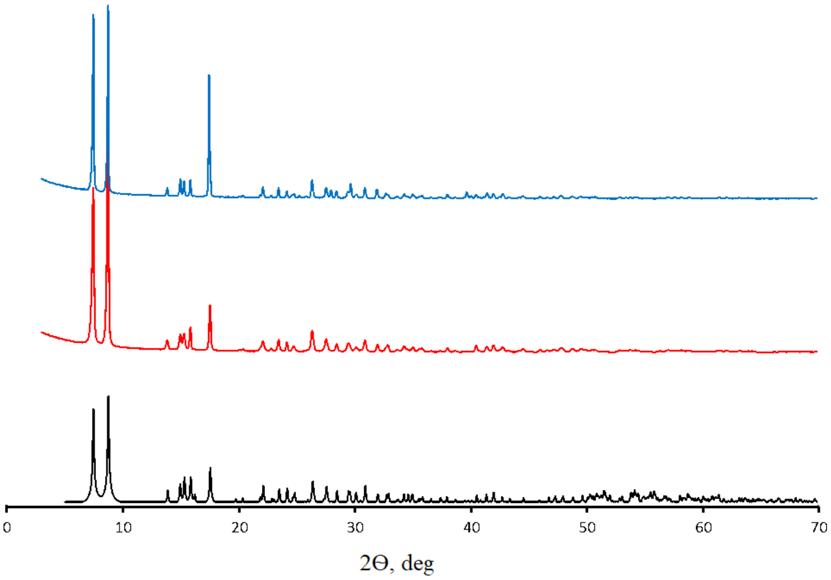

Figure S2. Shifted XRPD of the synthesized modified Zr-MOFs: MIL-140A-AcOH (red line), MIL-140A-AcOH-EDTA (blue line), simulated from cif [1] (black line).

The XRPD patterns of the obtained Zr-MOFs are consistent with the calculated structure.

Low-temperature nitrogen adsorption

The study by low-temperature nitrogen adsorption (BET) was carried out on an automated ASAP 2020MP system (Micromeritics). Nitrogen adsorption isotherms of Zr-MOFs are presented in Figure S3.

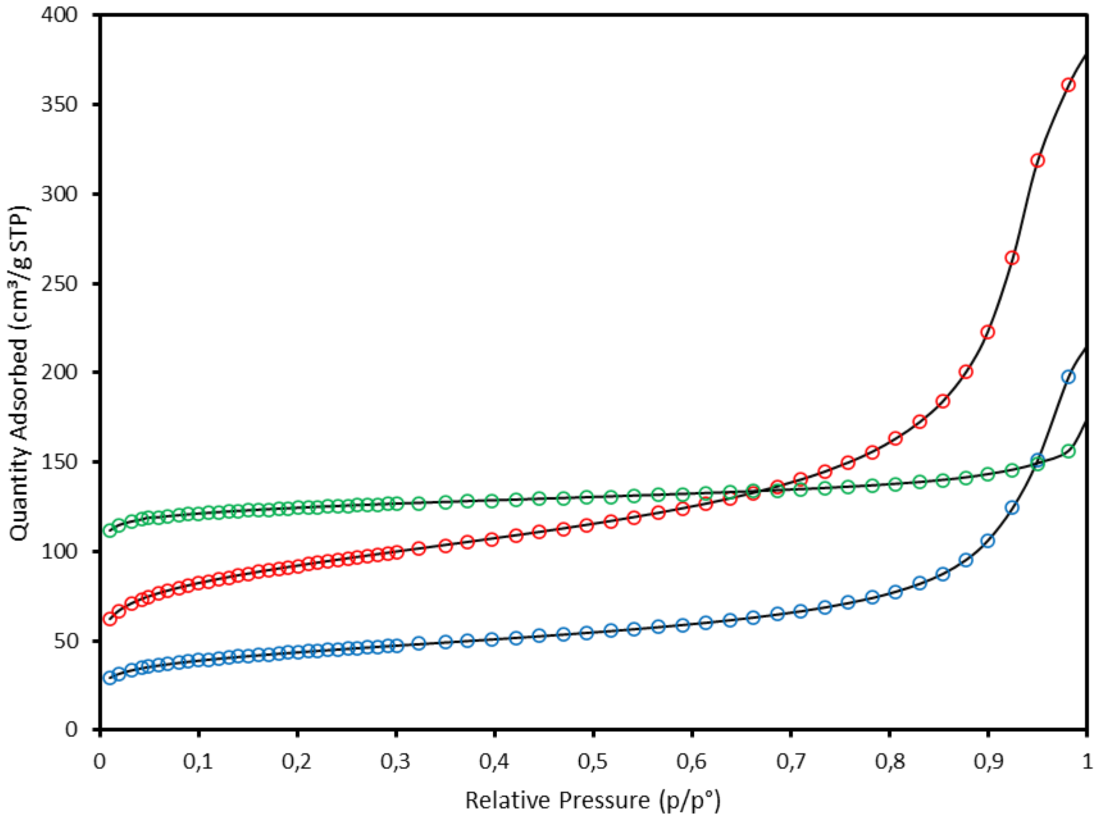

Figure S3. Low-temperature N2 adsorption isotherms: MIL-140A (green line), MIL-140A-AcOH (red line), MIL-140A-AcOH-EDTA (blue line).

The specific surface area was calculated by the Langmuir region of the isotherm dates and was equal to 493.4 ± 0.2 m²/g for MIL-140A, 568.0 ± 0.1 m²/g for MIL-140A-AcOH, and 529.3 ± 0.2 m²/g for MIL-140A-AcOH-EDTA. The resulting pore diameter of Zr-MOFs are 3.1, 4.4, and 3.5 Å for MIL-140A, MIL-140A-AcOH, and MIL-140A-AcOH-EDTA, respectively.

Scanning electron microscopy (SEM)

SEM images of the synthesized Zr-MOFs were obtained using a scanning electron microscope Hitachi S-3400N at 1 kV (Figure S4).

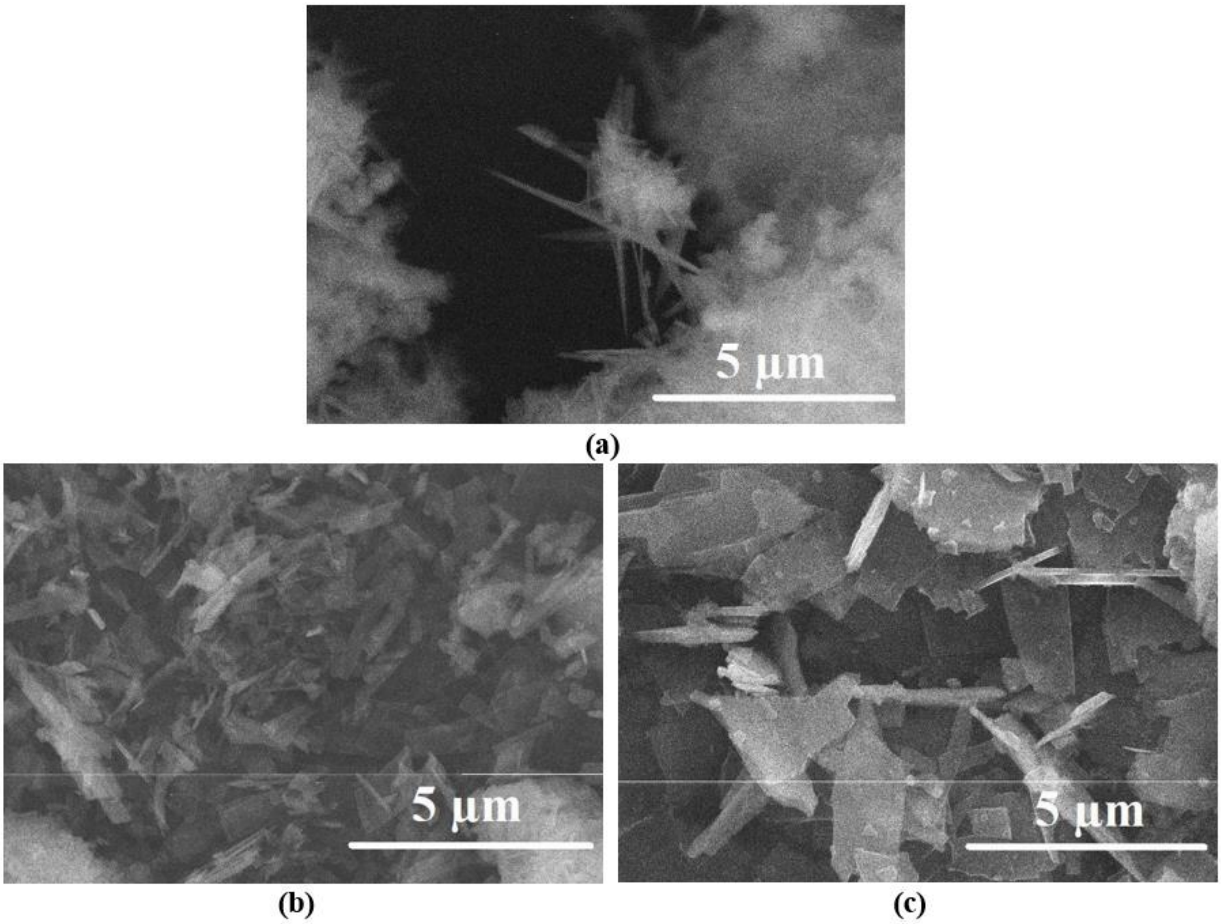

Figure S4. SEM images of the (**a**) MIL-140A, (**b**) MIL-140A-AcOH, and (**c**) MIL-140A-AcOH-EDTA.

The Zr-MOFs differ from each other by shape and size. Thus, unmodified MIL-140A is a narrow cylinder at least 5 μm long, while modified Zr-MOFs (MIL-140A-AcOH and MIL-140A-AcOH-EDTA) are mainly plates. The appearance of the powders differs from each other depending on the structure of the Zr-MOFs. Thus, MIL-140A is a narrow cylinder at least 5 μm long, while modified Zr-MOFs (MIL-140A-AcOH and MIL-140A-AcOH-EDTA) are mainly plates. The different structure of modifiers has a different effect on the membrane properties, reflected in both physicochemical and transport characteristics of the developed PVA/Zr-MOFs membranes.

References

1. Guillerm, V.; Ragon, F.; Dan-Hardi, M.; Devic, T.; Vishnuvarthan, M.; Campo, B.; Vimont, A.; Clet, G.; Yang, Q.; Maurin, G.; et al. CCDC 905026: Experimental Crystal Structure Determination, 2014. *Angew. Chem. Int. Ed.* **2012**, *51*, 9267. https://doi.org/10.5517/cczcrdg
